# Short Inverted Repeats as Mutational Hotspots and Putative Drivers of Genome Instability in Osteosarcoma

**DOI:** 10.3390/genes16101202

**Published:** 2025-10-14

**Authors:** Minghua Li, Chun Liang

**Affiliations:** 1 Department of Computational Medicine and Bioinformatics, University of Michigan, Ann Arbor, MI 48109, USA; 2 Department of Biology, Miami University, Oxford, OH 45056, USA; liangc@miamioh.edu

**Keywords:** short inverted repeat, SNV, INDEL, breakpoint, genome instability, osteosarcoma

## Abstract

**Background/Objectives**: Short inverted repeats (SIRs) are abundant DNA motifs capable of forming secondary structures, such as hairpins and cruciforms, that can induce genome instability. However, their mutational consequences in cancer, particularly in osteosarcoma (OS), remain largely unexplored. **Methods**: In this study, we systematically identified over 5.2 million SIRs in the human genome and analyzed their mutational patterns across six common cancer types. **Results**: We found that increased small insertion and deletion (INDEL) density within SIR spacer regions represents a consistent feature across cancers, whereas elevated single nucleotide variant (SNV) and structural breakpoint density is cancer-type specific. Integrating whole-genome sequencing data from 13 OS patients, we found that both SNVs and INDELs are significantly enriched within SIR spacer regions in OS. Notably, genomic regions with higher SIR density tend to accumulate more somatic mutations, suggesting a link between SIR abundance and local genome instability. SIR-associated mutations frequently occur in oncogenes and tumor suppressor genes, including *TP53*, *NFATC2*, *MECOM*, *LRP1B*, *RB1*, *CNTNAP2*, and *PTPRD*, as well as in long non-coding RNAs. Mutational signature analysis further suggests that defective DNA mismatch repair and homologous recombination may act in concert with SIR-induced DNA structural instability to drive OS development. **Conclusions**: Our findings highlight SIRs as mutational hotspots and potential drivers of osteosarcoma pathogenesis.

## 1. Introduction

Approximately 50–70% of the human genome comprises repetitive elements, including transposable elements, microsatellites, and inverted repeats (IRs) [1,2]. Despite their prevalence, our understanding of these repetitive elements remains limited, particularly regarding their composition and functionalities. IRs are a class of repetitive DNA sequences in which two arms are perfectly or imperfectly reverse complementary around a central spacer [3]. Based on length and base pairing status, IRs can be classified into long IRs (LIRs; >500 nucleotides [nt]) or short IRs (SIRs; <500 nt), as well as perfect IRs (perfectly matched arms) and imperfect IRs (containing mismatches between arms) [4,5]. The majority of IRs in the human genome are short (<100 nt) [6]. Palindromes represent a special case of IRs, in which the arms are reverse complementary without a spacer.

IRs can adopt secondary DNA structures such as hairpins in single-stranded DNA and cruciforms in double-stranded DNA, in which the arms hybridize to form a stem while the spacer remains unpaired [7,8,9,10]. Even relatively short IRs, such as those with a 7-nt arm, can form stable hairpins in vivo in yeast [11]. In vitro studies have utilized atomic force microscopy to observe that certain chromatin-associated enzymes bind cruciform structures formed by IRs as short as 106 nt [12]. Cruciform formation requires an IR arm length of at least 6 nt, regardless of whether the IR is perfect or imperfect [5,7,13]. Such secondary structures can serve as important binding sites for a variety of regulatory proteins, including restriction enzymes, transcriptional factors, and DNA repair and replication proteins [7,14]. For example, Emmerich et al. demonstrated that a 15-nt imperfect IR is essential for binding of the transcriptional regulator RegR [15].

The ability of IRs to form hairpins and cruciforms is closely linked to genome instability [5,16,17]. Hairpin structures form in single-stranded DNA, typically during DNA replication, transcription, or repair, when the DNA duplex becomes unwound [8,10,16]. Such hairpins can stall replication forks and generate double-strand breaks (DSBs), leading to insertions, deletions, or rearrangements [4,16,18]. Cruciform extrusion occurs when negative supercoiling accumulates behind DNA replication or transcription machinery, relieving torsional stress [16,19,20]. Cruciforms can be cleaved by structure-specific endonuclease, producing DNA nicks that are processed into hairpin-capped DSBs, which pose a threat to genome stability due to error-prone repair processing [16,20,21,22]. DSB repair occurs primarily through homologous recombination (HR), which uses sister chromatids as templates and is generally error-free, or non-homologous end-joining (NHEJ), which is active throughout the cell cycle but prone to errors due to its sequence-independent ligation mechanism [21,23,24,25]. In mammalian cells, insertion of a 29-nt cruciform-forming SIR increased mutation rates, including small insertions, deletions, and large deletions (>200 nt), by ~3-fold, suggesting that SIRs can directly stimulate DSB formation [4].

Genome instability is a hallmark of cancer, providing tumor cells with a selective growth advantage [26]. It manifests as small-scale variations such as single-nucleotide variants (SNVs), small insertions and deletions (INDELs), and microsatellite instability (MSI), as well as large-scale structure variations collectively termed gross chromosomal rearrangements (GCRs), which include gene amplifications, copy number variations (CNVs), chromothripsis, translocations, and inversions [27,28]. These alterations can disrupt caretaker genes (e.g., DNA damage checkpoint genes) or amplify oncogenes, thereby promoting tumorigenesis [29].

IR-associated genome instability has been observed in multiple cancers. In *HER2*-positive breast tumors, palindromic DNA sequences are enriched near amplified oncogenes such as *ERBB2* [30]. Experimental introduction of large SIRs in mammalian cells or yeast facilitates gene amplification through palindrome formation [31,32]. IR-mediated chromosomal translocations have also been identified in renal carcinoma [33], and recurrent mutations within SIRs have been detected [34,35].

Osteosarcoma (OS) is the most prevalent primary malignant bone tumor, predominantly affecting children and adolescents, with a notable occurrence rate among individuals aged over 60 years [36]. Metastasis constitutes the primary cause of death associated with OS, with a 5-year survival rate below 20% in metastatic cases [37]. Current OS treatments, including surgery, radiotherapy, and chemotherapy, remain inadequate [38]. Although *HER2* overexpression has been linked to lung metastasis in OS, targeted therapies have not significantly improved patient survival [39,40]. This underscores the urgent need to identify novel molecular drivers and therapeutic targets.

Given their abundance in the human genome, capacity to form secondary DNA structures, and mutagenic potential, SIRs are strong candidates for contributing to OS genome instability. However, the mutational landscape and functional consequences of SIRs in osteosarcoma remain poorly understood. A systematic characterization is therefore warranted, encompassing both perfect and imperfect SIRs, their distributions across protein-coding and non-coding regions, their associations with different forms of genome instability in OS, and the genes affected by SIR-associated mutations.

Several studies have explored SIR-related mutagenicity in other cancer types. For example, Zou et al. reported that the spacer regions of perfect SIRs in breast cancer exhibit a higher mutation density than matched control sequences [10]. However, genome instability patterns vary substantially across cancer types, each with distinct mutational signatures [41]. Notably, OS exhibits a higher incidence of chromothripsis events than breast cancer [42,43]. Furthermore, although both perfect and imperfect SIRs can form hairpin or cruciform structures, it remains unclear whether their mutational profiles are comparable. While most current studies focus on mutations in protein-coding genes, increasing evidence indicates that long non-coding RNAs (lncRNAs) play critical roles in diverse biological processes and cancer progression [44,45,46,47]. Investigating SIR-associated mutations in lncRNA genes may therefore reveal additional therapeutic opportunities.

In this study, we identified and characterized over 5.2 million SIRs in the human genome, including approximately 4.4 million perfect and 0.8 million imperfect SIRs. SIRs were unevenly distributed across chromosomes, preferentially located in the regions with lower GC content, and exhibited distinct densities between functional genomic categories—most notably, higher density in lncRNAs compared to mRNAs. We also analyzed SIR mutational patterns across six common cancer types (breast, liver, prostate, pancreas, lung, and skin cancer) and found consistently elevated INDEL density within SIR spacers, whereas increased SNV and structural breakpoint density were cancer-type specific.

Finally, using whole genome sequencing (WGS) data from 13 OS patients, we comprehensively profiled SIR-associated somatic mutation. Both SNV and INDEL densities were significantly higher within SIR spacers than in flanking regions. Regions with higher SIR density tended to accumulate more mutations, suggesting an association between SIR abundance and genome instability. SIR-associated mutations were frequently located in oncogenes and tumor suppressor genes (e.g., *TP53*, *NFATC2*, *MECOM*, *LRP1B*, *RB1*, *CNTNAP2*, *PTPRD*) as well as lncRNAs. Moreover, the mutation profiles of SIR-associated SNVs in OS resembled the catalogue of somatic mutations in cancer (COSMIC) [48] signatures indicative of defective DNA mismatch repair and double-strand break repair, suggesting potential synergy between SIR-induced structural instability and DNA repair deficiencies. Collectively, our findings unveil SIRs as mutational hotspots and potential drivers of OS pathogenesis.

## 2. Materials and Methods

### 2.1. Identification and Characterization of SIRs

We adopted Generic Repeat Finder (GRF) [5] v1.0.2 to identify SIRs in the human reference genome (hg38/GRCh38) [49], using the following criteria: spacer length of 4–8 nt, arm length of 6–25 nt, allowing at most one mismatch in the arms, and no INDELs permitted within arms. For mismatches, only SIRs with arm lengths > 9 nt were permitted to harbor mismatches. If multiple SIRs overlapped, we retained the longest one to minimize redundancy. The start position of the spacer was used to define the genomic coordinate of each SIR. SIRs were classified as perfect if their arms were perfectly complementary and as imperfect if mismatches were present.

SIR density, defined as the number of SIRs per million base pairs of sequence, was calculated across diverse functional and regulatory genomic features. Genomic annotations for lncRNAs and mRNAs, including exons, introns, 5′ UTRs, 3′ UTRs, coding sequence (CDS), and ±2 kb upstream/downstream regions, were obtained from GENCODE [50] via the UCSC Table Browser [51]. Candidate cis-Regulatory Elements (cCREs) were downloaded from ENCODE [52], including promoter-like, enhancer-like, histone modification (H3K4me3 and H3K27ac), and CTCF binding sites. Overlaps between SIRs and genomic features were identified using BEDTools [53] v2.31.0.

### 2.2. COSMIC Datasets

We extracted somatic SNVs and INDELs (both coding and non-coding) from the Genome Screen Mutant v98 and Non-Coding Variants v98 datasets of COSMIC [48]. Variants were grouped by cancer type, and only those from breast, liver, lung, prostate, skin and pancreas cancers were retained. Somatic breakpoints data for these six cancer types were also retrieved from COSMIC, covering intra- and inter-chromosomal rearrangements, tandem duplications, deletions, inversions and other categories. BEDTools [53] was used to identify overlaps between SIR coordinates and COSMIC somatic mutation data (SNVs, INDELs, and breakpoints).

### 2.3. Whole Genome Sequencing Analysis

Whole genome sequencing (WGS) data from 13 osteosarcoma (OS) patients (tumor-normal pairs; total 26 datasets) were obtained from dbGaP (accession: phs000699.v1.p1), along with associated clinical data (age at diagnosis, gender, metastatic status, survival time, etc.). All datasets consisted of pair-ended reads generated on the Illumina HiSeq 2000 platform.

Somatic SNVs and INDELs were called using the GATK [54] best practice pipelines. Raw pair-ended sequencing reads were subject to quality control using FastQC [55] v0.11.9, followed by preprocessing steps that involved the removal of adapters, low-quality ends, and reads containing poly-N bases via Trimmomatic [56] v0.39. Clean reads were then mapped to the hg38/GRCh38 build of the human reference genome using BWA [57] v0.7.17. We applied Picard MarkDuplicates [54] v2.27.4 to perform duplicate marking to reduce putative biases introduced by data generation steps such as PCR amplification. The BaseRecalibrator module of GATK v4.2.6.1 was used to detect and correct systematic bias introduced by library preparation and instrumentation defects. Somatic SNVs and INDELs were detected using Mutect2 [58], followed by functional annotation with Funcotator [54]. Annotation resources from several databases, including GENCODE [50], COSMIC [48], and Genome Aggregation Database (gnomAD) [59], were used for functional annotation of somatic SNVs and INDELs.

Somatic breakpoint data for these patients were obtained from Perry et al. [60] and lifted over from hg19/GRCh37 to hg38/GRCh38 using UCSC LiftOver [51]. Breakpoints were annotated using the FuncotateSegments module of GATK v4.2.6.1 along with GENCODE [50,54].

BEDTools [53] was adopted to identify overlaps between SIR coordinates and mutation data from OS patients (SNVs, INDELs and breakpoints).

### 2.4. SIR Mutation Analysis

For both COSMIC and OS datasets, mutation densities were compared for entire SIR sequences, spacers, arms, and flanking control sequences (100 nt on each side). SNV density = (Number of SNVs)/(Sequence length); INDEL density = (Number of basepairs altered by INDELs)/(Sequence length). For breakpoint analysis, we applied a 100-nt sliding window (10-nt step) over each SIR and flanking regions (±500 or ±1000 nt), to calculate positional breakpoint density. Hypermutated SIRs were identified using a binomial test, comparing spacer mutation density to control sequences, with Benjamini–Hochberg (BH) FDR < 0.05.

### 2.5. Somatic Mutation Analysis

We analyzed SIR overlapping SNVs, INDELs, and breakpoints using Maftools [61] v2.14.0. The top mutated genes were cross-referenced with the COSMIC [48] Cancer Gene Census (v98) to identify enriched oncogenes and tumor suppressors. SNVs were categorized into six transition and transversion events, including C>A/G>T, C>G/G>C, C>T/G>A, T>A/A>T, T>C/A>G, and T>G/A>C. Each SNV category was further classified into 16 subtypes based on adjacent 5′ and 3′ bases surrounding the substituted base, resulting in 96 possible trinucleotide contexts [41]. Mutational signature analysis was performed using Maftools [61] to extract mutational signatures and matched them against COSMIC [48] reference signatures.

Variant allele frequency (VAF) analysis was conducted using Maftools [61] to calculate the fraction of sequencing reads that support the allele with a specific alteration relative to the overall reads in a specific genomic locus.

Gene co-occurrence and mutual exclusivity were assessed using Fisher’s exact test on a 2 × 2 contingency table including frequencies of samples altered or unaltered for the gene pairs. Clinical enrichment was evaluated via a similar approach that performed Fisher’s exact test on the contingency tables including frequencies of samples altered or unaltered for every gene and clinical variables. Kaplan–Meier survival analysis [62] was used to compare survival between patients with and without SIR mutations in specific genes. Gene-drug interactions were identified by querying DGIdb [63] and determining druggable categories. Maftools [61] was used to map SIR-mutated genes against TCGA [64] oncogenic signaling pathways to determine altered oncogenic signaling pathways and genes of relevance.

### 2.6. Statistics and Visualization

Unless otherwise stated, results are expressed as mean ± s.e.m. Linear regression was performed using the R “lm” function. Statistical tests were performed using R v4.2.0 and the SciPy [65] v1.13.1 library in Python v3.12.4. In particular, binomial tests with BH correction were conducted using SciPy’s “binom_test” and “fdrcorrection”.

Visualizations were generated using the Matplotlib [66] library in Python, base R plotting functions, Maftools [61], and Circos [67] v0.69.9. Mutation plots were primarily produced with Maftools; the genome-wide breakpoint plot was generated using Circos.

## 3. Results

### 3.1. Identification and Characterization of SIRs in the Human Genome

**Figure 1:**
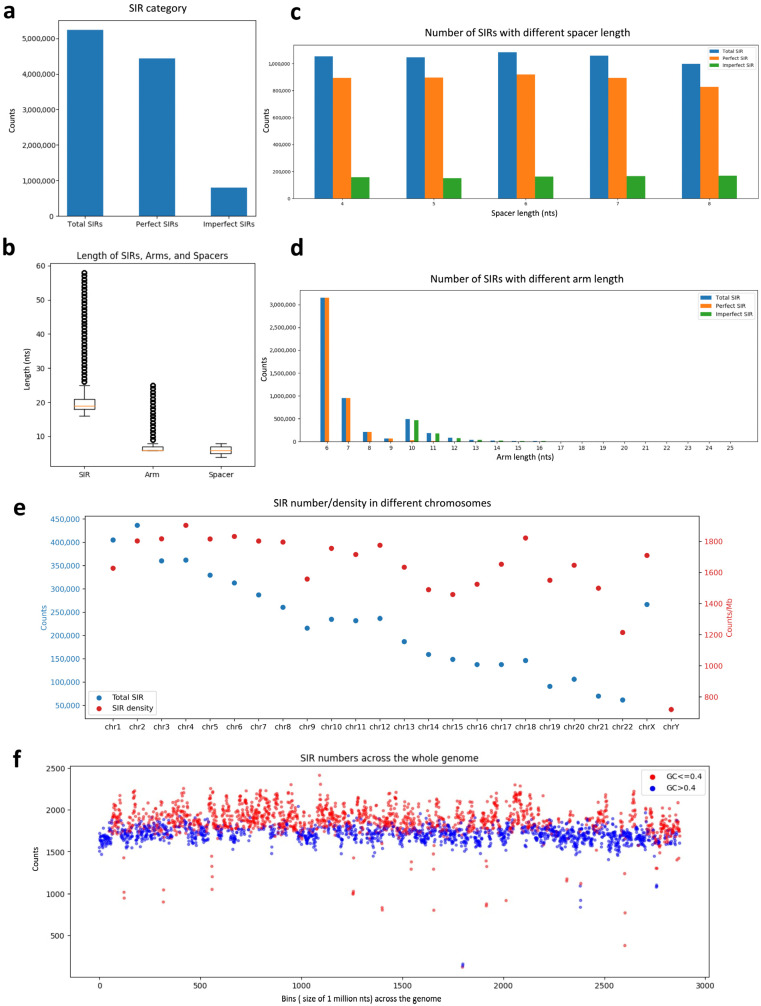
Characterization of Short Inverted Repeats (SIRs) in the Human Genome. (**a**) Distribution of perfect and imperfect SIRs across the human genome; (**b**) Mean length of SIRs, spacers, and arms; (**c**) Frequency of SIRs with different spacer lengths; (**d**) Frequency of SIRs with different arm lengths; (**e**) Chromosomal distribution of SIR abundance (blue, left y-axis) and SIR density (red, right y-axis); (**f**) Comparison of SIR density in genomic regions with low GC content (≤0.4) versus high GC content (>0.4).

We identified 5,243,326 SIRs in the human genome, comprising 4,434,473 perfect SIRs and 808,853 imperfect SIRs (Figure 1a). The average length of all SIRs was 20.22 ± 4.18 nts (standard deviation), with an average spacer length of 5.98 ± 1.4 nts and an average arm length of 7.12 ± 1.96 nts (Figure 1b). Across spacer lengths, SIR frequencies were relatively uniform, ranging from 997,334 (8-nt spacer) to 1,083,786 (6-nt spacer) (Figure 1c). Interestingly, perfect SIRs with an 8-nt spacer were the least abundant (828,386), whereas imperfect SIRs with an 8-nt spacer were the most abundant (168,948) among all imperfect SIRs, suggesting a preferential formation of imperfect SIRs at this spacer length.

Regarding arm length, SIR abundance decreased as the arm length increased, with 6-nt arms being the most common (3,155,834) (Figure 1d). Imperfect SIRs were only allowed when the arm length exceeded 9 nt, resulting in a pronounced peak of 466,900 imperfect SIRs with a 10-nt arm, followed by a gradual decline as the arm length increased. We also examined the frequency distribution of individual spacer sequences (Table 1). For example, although the total number of SIRs with a 4-nt spacer (1,054,338) was similar to those with a 7-nt spacer (1,059,226), the most frequent 4-nt spacer AGTG occurred 26,165 times, far more often than the most frequent 7-nt spacer TATATCT (9676 occurrences). SIR distribution varied substantially among chromosomes. Chromosome 4 had the highest SIR density (1902 SIRs/Mb), whereas chromosome Y had the lowest (720 SIRs/Mb) (Figure 1e). Although chromosome 2 contained the largest total number of SIRs, it did not have the highest density, indicating variation in chromosomal SIR distribution independent of chromosome size.

We then assessed the relationship between SIR density and guanine-cytosine (GC) content. The mean GC content of the human genome is approximately 0.4. Regions with low GC content (≤0.4) exhibited significantly higher SIR density than high GC content regions (>0.4) (*p*-value = 1.25 × 10^−199^, two-tailed Student’s *t*-test; Figure 1f). This trend was consistent for imperfect SIRs (*p*-value = 1.19 × 10^−202^), indicating a strong preference of SIRs for AT-rich genomic regions.

### 3.2. Distribution of SIRs in Functional Genomic Regions

**Figure 2:**
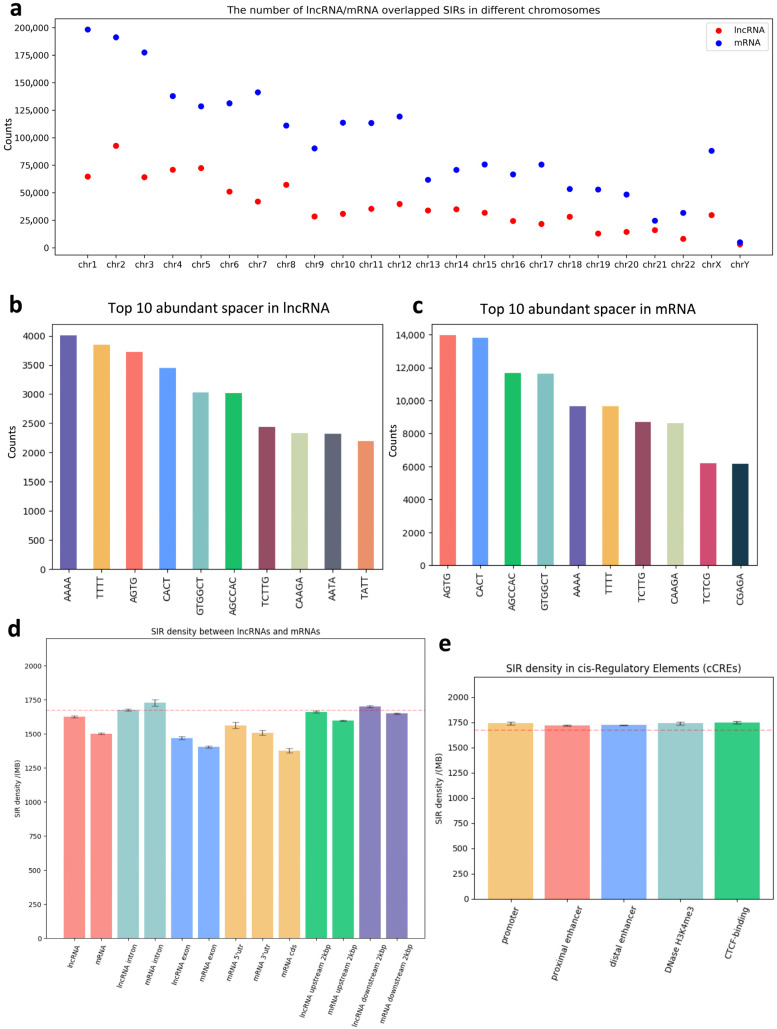
Distribution and sequence features of SIRs across functional genomic regions. (**a**) Chromosomal distribution of SIRs within protein-coding (mRNA) and long non-coding RNA (lncRNA) genes. The y-axis represents the number of SIRs per chromosome; (**b**) Top 10 most frequent spacer sequences in lncRNA-associated SIRs; (**c**) Top 10 most frequent spacer sequences in mRNA-associated SIRs; (**d**) SIR density (SIRs per million basepairs) in lncRNA, mRNA, and their flanking genomic regions. CDS, coding sequence; UTR, untranslated region. The red dashed line represents the average SIR density in the human genome; (**e**) SIR density across candidate cis-regulatory elements (cCREs) annotated by the ENCODE project, including promoters, proximal enhancers, distal enhancers, DNase H3K4me3 sites, and CTCF-binding regions. All cCRE types displayed higher SIR density compared to background genomic regions, but no significant differences were observed among the cCRE categories.

We examined the distribution of SIRs across different functional genomic regions. In total, we identified 2,309,610 SIRs within protein-coding genes and 909,254 within lncRNAs. While protein-coding genes contain more SIRs overall, our analysis revealed that the majority of these SIRs are located within intronic regions rather than in exons (Table S1). Among SIRs in protein-coding genes, 1,963,062 were perfect and 346,548 were imperfect, whereas in lncRNAs, 765,427 were perfect and 143,827 were imperfect. The perfect-to-imperfect SIR ratio was slightly higher in protein-coding genes than in lncRNA genes (5.66 vs. 5.32). The chromosomal distribution of SIRs also differed between lncRNAs and mRNAs (Figure 2a). Chromosome 1 contained the largest number of mRNA-associated SIRs, whereas chromosome 2 harbored the highest number of lncRNA-associated SIRs.

Spacer sequence preferences varied between SIRs located in coding and non-coding regions. As shown in Figure 2b,c, the most frequent spacers within lncRNA-associated SIRs were AAAA and TTTT, whereas these spacers ranked lower in mRNA-associated SIRs, suggesting distinct spacer composition biases between the two gene types. We further compared SIR density between lncRNAs, mRNAs, and their flanking genomic sequences (Figure 2d). SIR density was significantly higher in lncRNAs than in mRNAs (*p*-value = 5 × 10^−49^, two-tailed Student’s *t*-test). Within mRNAs, the coding sequence (CDS) exhibited the lowest SIR density, whereas introns showed the highest. For both lncRNAs and mRNAs, exon regions consistently had lower SIR density compared to intronic and flanking (upstream or downstream) regions.

Finally, we assessed SIR density across candidate cis-Regulatory Elements (cCREs) annotated by the ENCODE [52] project, including promoters, proximal enhancers, distal enhancers, DNase H3K4me3 sites, and CTCF binding regions (Figure 2e). All cCRE categories exhibited higher SIR density than background genomic regions; however, no significant differences were observed among the different cCRE types.

### 3.3. SIR-Associated Mutational Patterns Across Multiple Cancer Types

**Figure 3:**
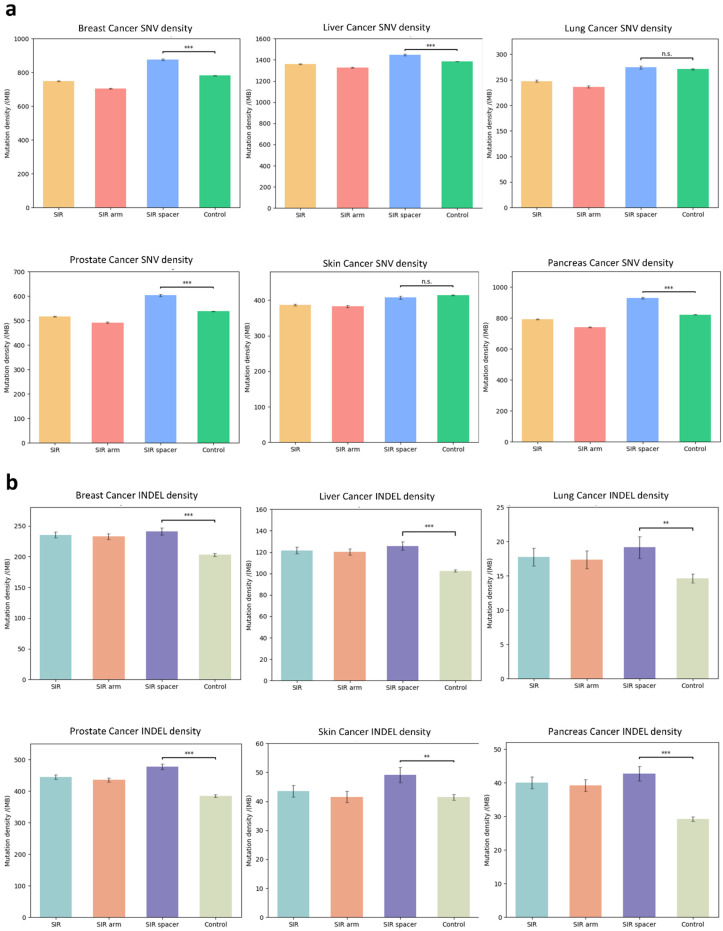
SIR-associated SNV and INDEL enrichment across multiple cancer types. (**a**) Comparison of single-nucleotide variant (SNV) density within short inverted repeat (SIR) spacer regions versus ±100 nt flanking control sequences in six cancer types (breast, liver, lung, prostate, skin, and pancreas) from the COSMIC database. Breast, liver, prostate, and pancreas cancers showed significant enrichment of SNVs within spacers, whereas lung and skin cancers did not; (**b**) Comparison of insertion and deletion (INDEL) density within SIR spacers versus flanking control sequences for the same six cancer types. INDEL density was significantly higher within spacers across all cancer types examined. SNV density was calculated as the number of SNVs per sequence length; INDEL density was calculated as the number of base pairs altered by INDELs per sequence length. Statistical significance was assessed using two-tailed Student’s *t*-tests. *p*-value < 0.05 was considered significant (** *p* < 0.01, *** *p* < 0.001, n.s. *p* ≥ 0.05).

To investigate whether SIRs exhibit distinct mutational patterns in cancer, we analyzed mutation profiles from six common cancer types: breast, liver, lung, prostate, skin, and pancreatic cancers. We compared the mutation density within SIR spacer regions against flanking sequences located upstream and downstream. The enrichment of SNVs within SIR spacers varied among cancer types. For example, breast, liver, prostate, and pancreas cancers exhibited a significant increase in SNV density within spacers, whereas lung and skin cancers showed no significant difference relative to flanking control sequences (Figure 3a). In contrast, INDELs were significantly enriched within SIR spacers across all cancer types examined (Figure 3b), indicating that spacer regions are preferential sites for insertions and deletions.

**Figure 4:**
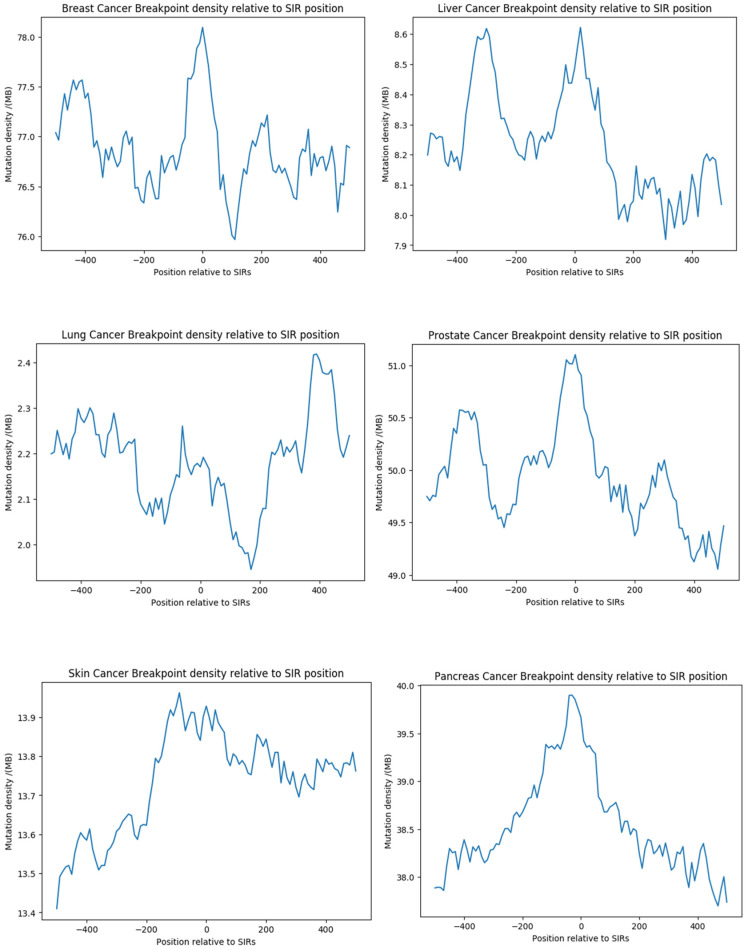
SIR-associated breakpoint enrichment across multiple cancer types. Average breakpoint density was profiled within SIRs and their flanking sequences (±500 nt) across six cancer types (breast, liver, lung, prostate, skin, and pancreas). Breast, prostate, skin, and pancreas cancers showed a pronounced peak at the SIR center, indicating breakpoint enrichment. Liver cancer displayed both a central peak and an additional peak on the left arm. In lung cancer, breakpoint density was elevated within SIRs relative to flanks only within ±200 nt. These patterns highlight cancer-specific genomic instability signatures associated with SIRs.

We also examined structural breakpoints in relation to SIRs. Enrichment patterns were cancer-type specific: breast, prostate, skin, and pancreas cancers exhibited a notable peak at the center of SIRs, where the average breakpoint density was higher than in their flanking sequences; liver cancer also displayed a peak at SIRs, accompanied by an additional peak on the left arm; in lung cancer, the average breakpoint density was higher in SIRs than flanking sequences at within a ±200 nts range but not beyond (Figure 4). This heterogeneity suggests that while SIR-associated INDELs represent a universal mutational feature across cancers, SNV and breakpoint enrichment within SIRs are influenced by cancer-specific genomic instability mechanisms.

### 3.4. Mutation Profiles of SNVs and INDELs Within SIRs in Osteosarcoma

**Figure 5:**
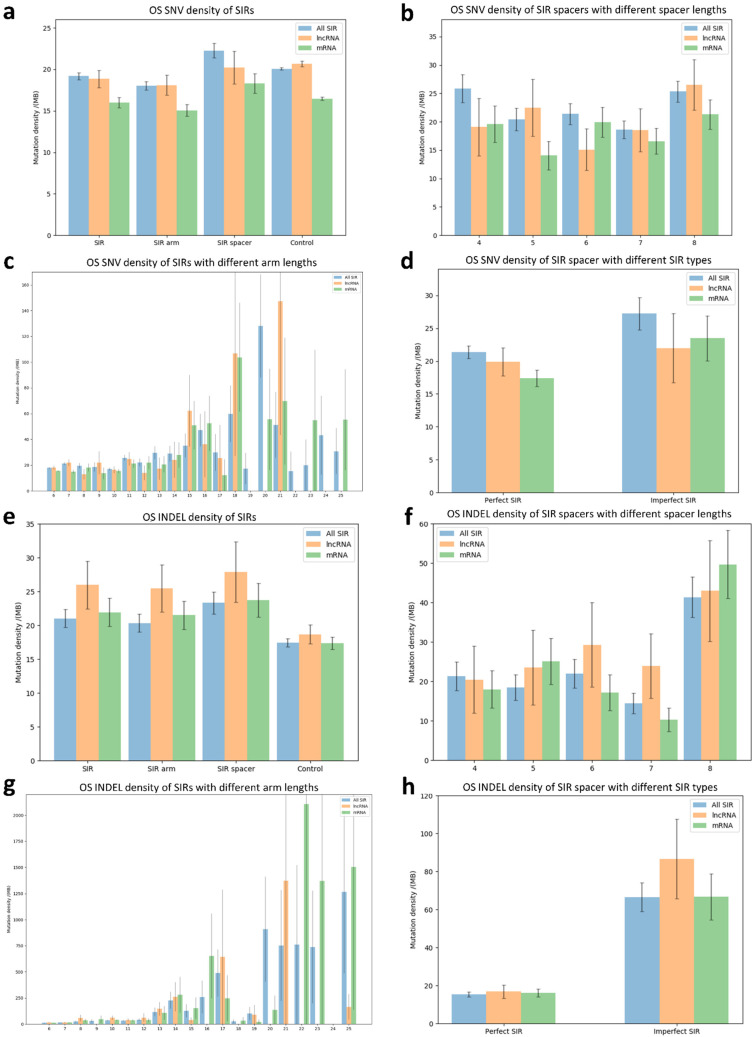
Mutation profiles of SNVs and INDELs within SIRs in osteosarcoma (OS). (**a**) Comparison of SNV density within SIRs, arms, spacers, and their flanking control sequences (100-nt); (**b**) SNV density within SIR spacers of different spacer lengths; (**c**) SNV density within SIRs with different arm lengths; (**d**) Comparison of SNV density within SIR spacers of perfect and imperfect SIRs; (**e**) Comparison of INDEL density within SIRs, arms, spacers, and their flanking control sequences (100-nt); (**f**) INDEL density within SIR spacers of different spacer lengths; (**g**) INDEL density within SIRs with different arm lengths; (**h**) Comparison of INDEL density within SIR spacers of perfect and imperfect SIRs.

We identified 58,773 somatic SNVs from the WGS data of 13 OS patients, of which 2078 were located within SIR regions. As depicted in Figure 5a, the SNV density within SIR spacers was significantly higher than in flanking control sequences (*p*-value = 1.3 × 10^−2^, two-tailed Student’s *t*-test). However, when stratified by gene type, neither lncRNA-associated nor mRNA-associated SIR spacers showed a significant increase in SNV densities compared with their respective controls. Moreover, no significant difference in SNV density was detected between lncRNA and mRNA-associated SIR spacers.

We next assessed whether SNV density varied with spacer or arm length. SIR spacers of different lengths exhibited significantly different SNV densities (*p*-value = 3.64 × 10^−2^, one-way ANOVA; Figure 5b), suggesting a length-dependent effect. Similarly, SNV density differed significantly across SIRs with varying arm lengths (*p*-value = 3.31 × 10^−7^, one-way ANOVA; Figure 5c). Perfect SIRs exhibited significantly different SNV densities compared with imperfect SIRs (*p*-value = 1.6 × 10^−2^, two-tailed Student’s *t*-test; Figure 5d). However, since mismatches were only permitted in SIRs with arm lengths > 9 nt, we repeated the analysis in this subset and found no significant difference, indicating that the observed difference was largely attributable to arm length rather than SIR type.

Regarding INDELs, we identified 17,482 somatic INDELs in OS, of which 870 overlapped SIR regions. INDEL density within SIR spacers was significantly higher than in control sequences (*p*-value = 8.29 × 10^−4^, two-tailed Student’s *t*-test; Figure 5e). This enrichment was observed in both lncRNA-associated (*p*-value = 4.93 × 10^−2^) and mRNA-associated (*p*-value = 1.71 × 10^−2^) SIR spacers relative to controls. Spacer length was strongly associated with INDEL density (*p*-value = 3.93 × 10^−6^, one-way ANOVA; Figure 5f), with 8-nt spacers exhibiting the highest density. Interestingly, spacer length-dependent INDEL variation was significant in mRNA-associated SIRs (*p*-value = 8.44 × 10^−6^) but not in lncRNA-associated SIRs, suggesting potential different mutational dynamics between coding and non-coding regions. Arm length was also significantly associated with INDEL density (*p*-value = 5.04 × 10^−212^, one-way ANOVA; Figure 5g), although the Pearson correlation coefficient indicated only a weak linear relationship (R = 8.76 × 10^−3^). Finally, spacers of perfect SIRs exhibited significantly different INDEL densities compared with spacers of imperfect SIRs (*p*-value = 6.8 × 10^−29^, two-tailed Student’s *t*-test; Figure 5h). As with SNVs, this difference disappeared when restricting analysis to SIRs with arm lengths > 9 nts, indicating that INDEL enrichment is independent of SIR type.

### 3.5. Relationship Between SNV Density, INDEL Density, and SIR Density

**Figure 6:**
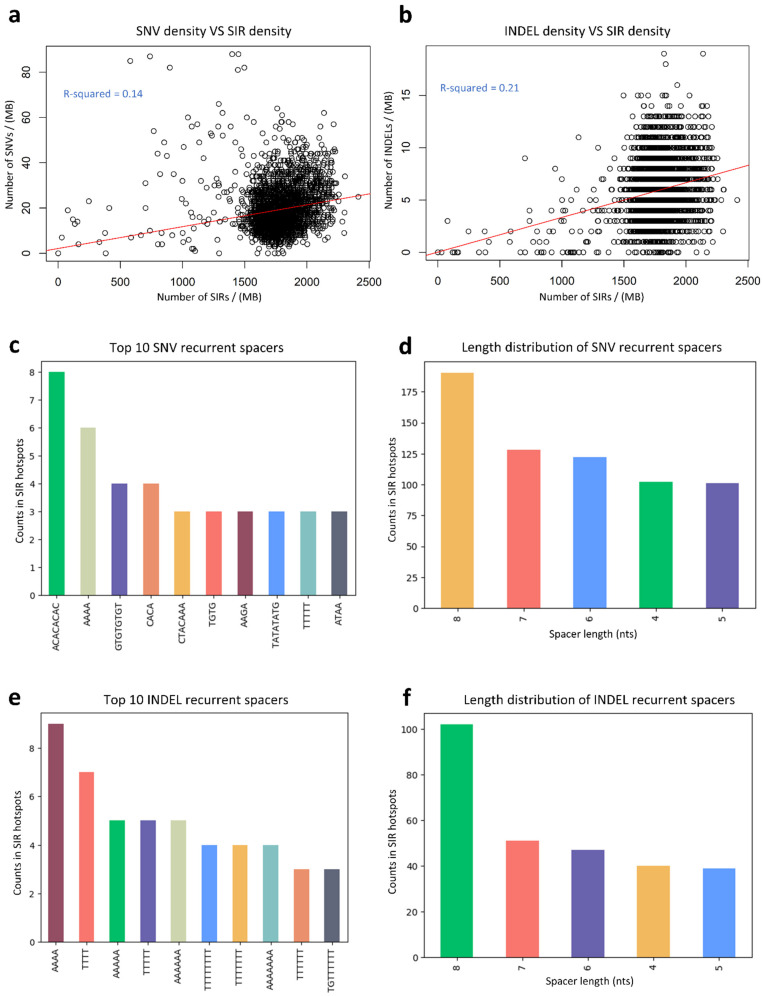
Relationship between SIR density and mutation density, and characterization of hypermutated SIRs. (**a**) Linear regression analysis (SIR–SNV model) showing the association between SNV density and SIR density across 1 Mb genomic bins. Six bins with SNV densities > 100/Mb were excluded as potential outliers; (**b**) Linear regression analysis (SIR–INDEL model) showing the association between INDEL density and SIR density across 1 Mb genomic bins; (**c**) Representative recurrent spacer sequences among hypermutated SIRs with significantly elevated SNV densities within spacers compared to control sequences; (**d**) Distribution of spacer lengths among hypermutated SIRs identified by SNV density, showing 8-nt spacers as the most enriched category; (**e**) Representative recurrent spacer sequences among hypermutated SIRs with significantly elevated INDEL densities within spacers compared to controls; (**f**) Distribution of spacer lengths among hypermutated SIRs identified by INDEL density, showing 8-nt spacers as the most enriched category.

To examine the relationship between SNV density and SIR density, we divided the genome into non-overlapping 1 Mb bins and applied a linear regression model (SIR-SNV model; Figure 6a). Six bins with SNV density greater than 100/Mb were excluded as potential outliers. The fitted model yielded an intercept of 2.16 (SE = 0.73) and a slope of 0.0096 (SE = 0.0004), with a slope *p*-value < 2 × 10^−16^, indicating a significant positive association between SIR density and SNV density. However, the *R^2^* value was 0.14, suggesting that only 14% of the variation in SNV density can be explained by SIR density.

We then applied a similar regression (SIR-INDEL model) to assess the association between SIR density and INDEL density (Figure 6b). This model produced an intercept of 0.032 (SE = 0.20) and a slope of 0.0033 (SE = 0.00011), with a slope *p*-value < 2 × 10^−16^ and *R*^2^ = 0.21. The higher *R*^2^ compared to the SIR-SNV model indicates that SIR density is more strongly correlated with INDEL density than with SNV density.

We identified 643 hypermutated SIRs with significantly higher SNV densities within their spacers than in flanking control sequences (Table S2). Several hypermutated spacers recurred across multiple SIR loci (Figure 6c). For example, the low-complexity spacer ‘ACACACAC’ was hypermutated in eight SIRs across chromosomes 1, 3, 4, 5, and 12, while the higher-complexity spacer ‘CTACAAA’ was hypermutated in three SIRs on chromosomes 2, 6, and X. Most hypermutated SIRs had an 8-nt spacer, approximately 1.5 times more frequent than those with a 7-nt spacer (Figure 6d).

Similarly, 279 hypermutated SIRs exhibited significantly higher INDEL densities within spacers than in flanking controls (Table S3). Recurrent hypermutated spacers were predominantly low-complexity sequences such as ‘AAAA’ and ‘TTTT’, suggesting that low-complexity spacers may be particularly prone to INDEL formation (Figure 6e). The 8-nt spacer length category again dominated, with twice as many hypermutated SIRs as the 7-nt category (Figure 6f).

### 3.6. Analysis of SIR-Associated SNVs and INDELs in Osteosarcoma

**Figure 7:**
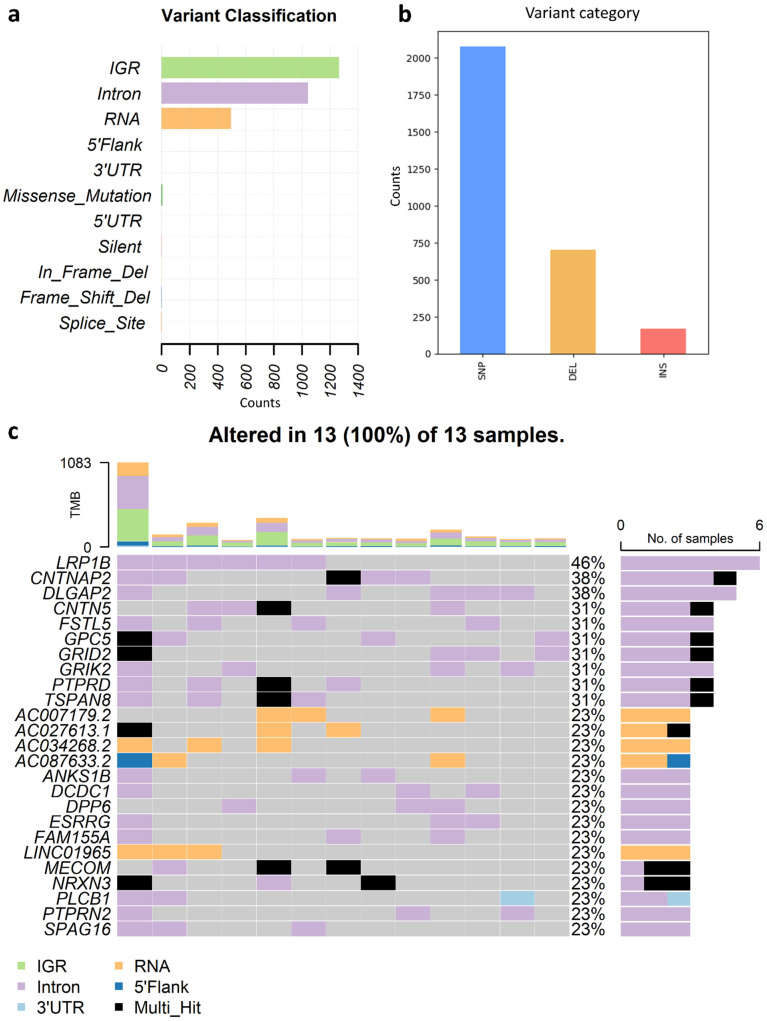
Genomic distribution and gene-level profiles of SIR-associated mutations in osteosarcoma (OS). (**a**) Genomic distribution of SIR-associated SNVs and INDELs across functional categories, including intergenic regions (IGRs), introns, non-coding RNA loci, and protein-coding regions; (**b**) Proportions of SIR-associated SNVs versus INDELs, with INDELs further classified into insertions and deletions; (**c**) Top 25 genes most frequently altered by SIR-associated SNVs or INDELs across 13 OS patients. Bars represent the number of patients with mutations in each gene.

We next performed a detailed characterization of SIR-associated SNVs and INDELs and examined the genes affected by these mutations in OS. Most SIR-associated variants localized to intergenic regions (IGRs), introns, and non-coding RNA loci, with fewer events in protein-coding regions (Figure 7a), suggesting greater mutability of SIRs in non-coding genomic compartments. Across the 13 OS genomes, SIR-associated SNVs outnumbered INDELs, with deletions occurring more frequently than insertions (Figure 7b).

Among the top 25 most frequently mutated genes (Figure 7c), LDL receptor-related protein 1B (*LRP1B*) was the most frequently altered, carrying predominantly intronic mutations in 6/13 patients. Annotated as a tumor suppressor in the COSMIC [48] database, *LRP1B* is also frequently mutated in chronic lymphatic leukemia, esophageal squamous cell carcinoma, ovarian, and urothelial cancers. *CNTNAP2* and *DLGAP2* ranked second, altered in 5/13 patients. *CNTNAP2*, another tumor suppressor, is recurrently mutated in glioma and melanoma as annotated in COSMIC. Additional tumor suppressors, including *GPC5* and *PTPRD*, also harbored SIR-associated mutations. Notably, the oncogene *MECOM*, implicated in acute myeloid leukemia and other hematologic malignancies [68,69], was among the top 25 genes, with mutations concentrated in SIRs. Five lncRNA genes (*AC007179.2*, *AC027613.1*, *AC034268.2*, *AC087633.2*, and *LINC01965*) were also recurrently mutated.

**Figure 8:**
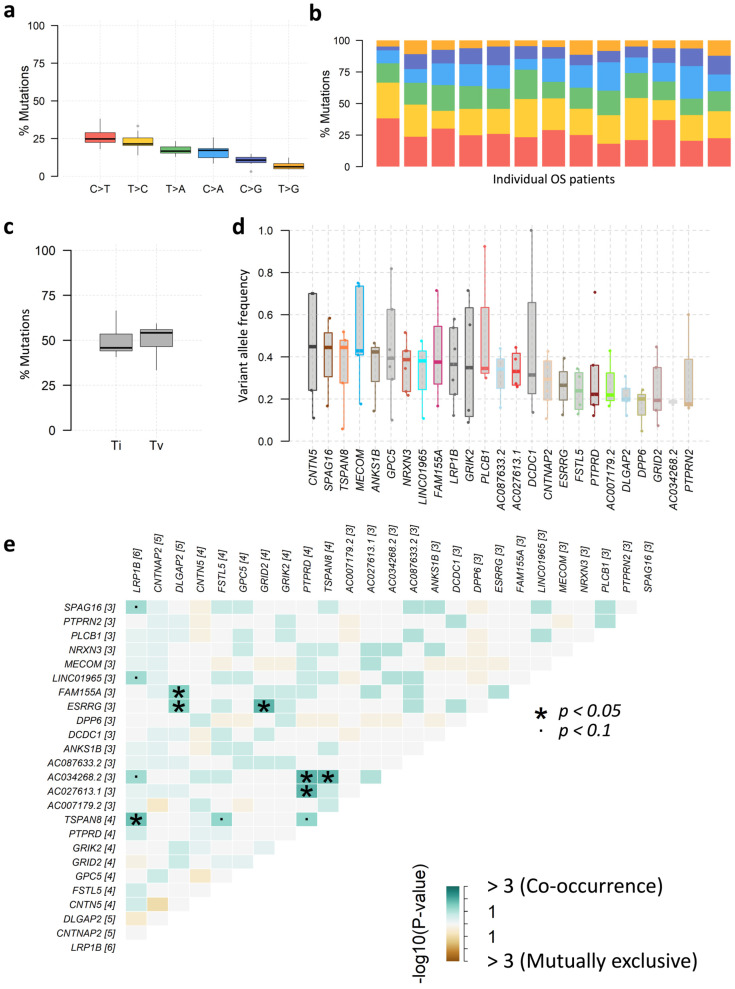
Mutational characteristics of SIR-associated SNVs and INDELs in osteosarcoma (OS). (**a**) Distribution of six SNV substitution classes: C>A/G>T, C>G/G>C, C>T/G>A, T>A/A>T, T>C/A>G, and T>G/A>C; (**b**) Stacked bar plots showing the composition of SNV classes per OS patient; (**c**) Overall proportions of transitions versus transversions in SIR-associated SNVs; (**d**) Variant allele frequency (VAF) distribution for the top 25 most frequently mutated genes containing SIR-associated mutations; (**e**) Co-occurrence and mutual exclusivity network among the top 25 mutated genes (Fisher’s exact test).

SNVs within SIRs can be classified into six transition and transversion events: C>A/G>T (conversions between the nucleotide base C and A, or between G and T), C>G/G>C, C>T/G>A, T>A/A>T, T>C/A>G, and T>G/A>C. We observed that C>T/G>A transitions were the most prevalent (555 SNVs), while T>G/A>C transversions were the least frequent (150 SNVs) (Figure 8a,b). The overall proportions of transitions and transversions were similar (Figure 8c). Variant allele frequency (VAF) analysis indicated that nearly all of the top 25 mutated genes had a median VAF above 20%, with MECOM exceeding 40% (Figure 8d), suggesting clonal, potentially driver events.

Co-occurrence analysis revealed significant positive associations among several top genes (Figure 8e). For example, the lncRNA *AC034268.2* was co-mutated with *LRP1B* and *PTPRD*, while *LRP1B* mutations were co-mutated with *LINC01965*, and *PTPRD* was co-mutated with *AC027613.1*, suggesting potential regulatory interplay between lncRNA-associated SIRs and tumor suppressor genes.

**Figure 9:**
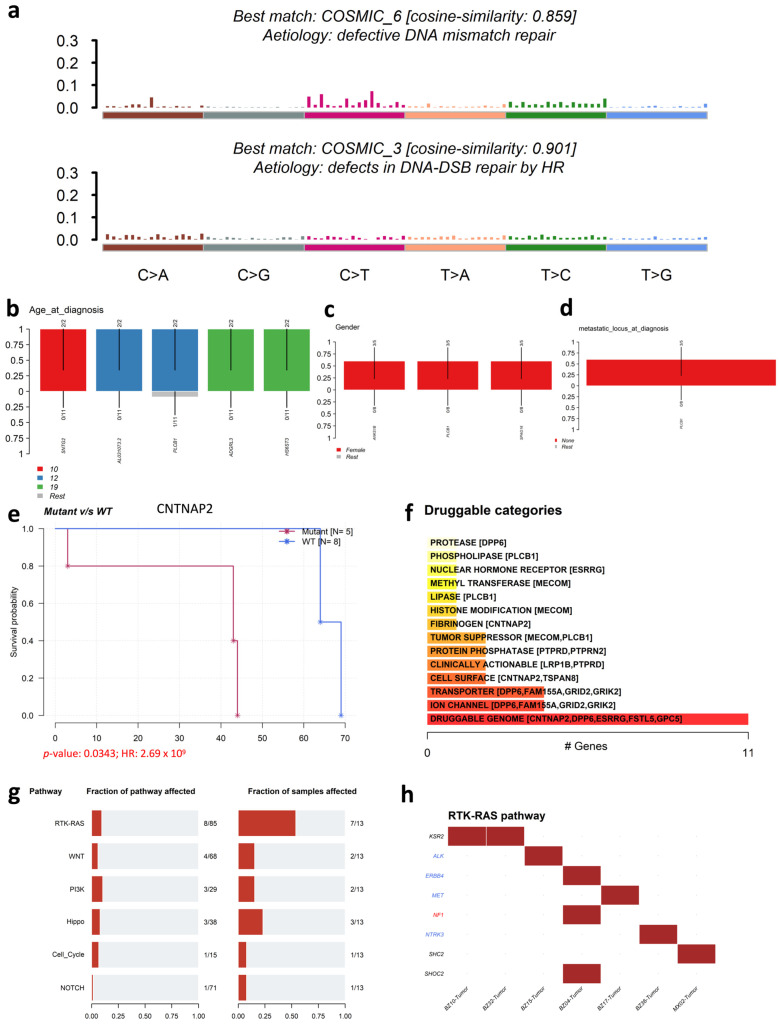
Mutational signatures and clinical associations of SIR-associated mutations in osteosarcoma (OS). (**a**) COSMIC mutational signature analysis of SIR-associated SNVs. Bar plot shows cosine similarity scores to top-matched signatures; signature 6 (defective DNA mismatch repair) and signature 3 (defects in DNA-DSB repair by HR) are highlighted; (**b**–**d**) Clinical enrichment analysis of SIR-associated mutations stratified by (**b**) age at diagnosis, (**c**) gender, and (**d**) metastatic status at diagnosis; (**e**) Kaplan–Meier survival curves for OS patients stratified by *CNTNAP2* mutation status; (**f**) DGIdb-based annotation of druggable mutated genes with SIR-associated mutations; (**g**) Distribution of altered oncogenic pathways in OS; (**h**) Frequency of SIR-associated mutations in individual genes within the RTK–RAS pathway. Gene names are color-coded: blue indicates oncogenes and red indicates tumor suppressor genes.

Mutational signature analysis demonstrated strong similarity to COSMIC signature 6 (defective DNA mismatch repair; cosine similarity 85.9%) and signature 3 (defects in DNA double-strand break repair by homologous recombination; cosine similarity of 90.1%) (Figure 9a), consistent with SIRs’ propensity to induce DNA double-strand breaks and their potential synergy with defective repair mechanisms in OS.

Clinical enrichment analysis identified subtype-specific associations (Figure 9b–d). For example, *SNTG2* mutations were enriched in patients diagnosed at age 10, *AL031073.2* and *PLCB1* at age 12, and *ADGRL3* and *HS6ST3* at age 19 (*p*-value < 0.05, Fisher’s exact test). *PLCB1* mutations were more common in females and enriched in non-metastatic cases at diagnosis (*p*-value < 0.05). *PLCB1*, recognized as an oncogene driving cancer progression, has been implicated in various cancers, including gastric cancer and cholangiocarcinoma [70,71]. *PLCB1* mutations appeared across all three clinical variables, suggesting a broader role in OS progression.

Kaplan–Meier analysis revealed that *CNTNAP2* mutation status was significantly associated with patient survival (Figure 9e). Analysis with Drug Gene Interaction Database (DGIdb) [63] indicated that most top mutated genes are druggable, with several (e.g., *DPP6*, *FAM155A*, *GRID2*, and *GRIK2*) targetable via ion channel inhibitors (Figure 9f).

Pathway analysis (Figure 9g–h) showed that the RTK-RAS oncogenic pathway was most frequently altered, with 8 genes mutated in 7/13 patients. Other altered pathways included WNT, PI3K, Hippo, cell cycle, and NOTCH, but at lower frequencies.

### 3.7. Analysis of SIR-Associated Breakpoints in Osteosarcoma

**Figure 10:**
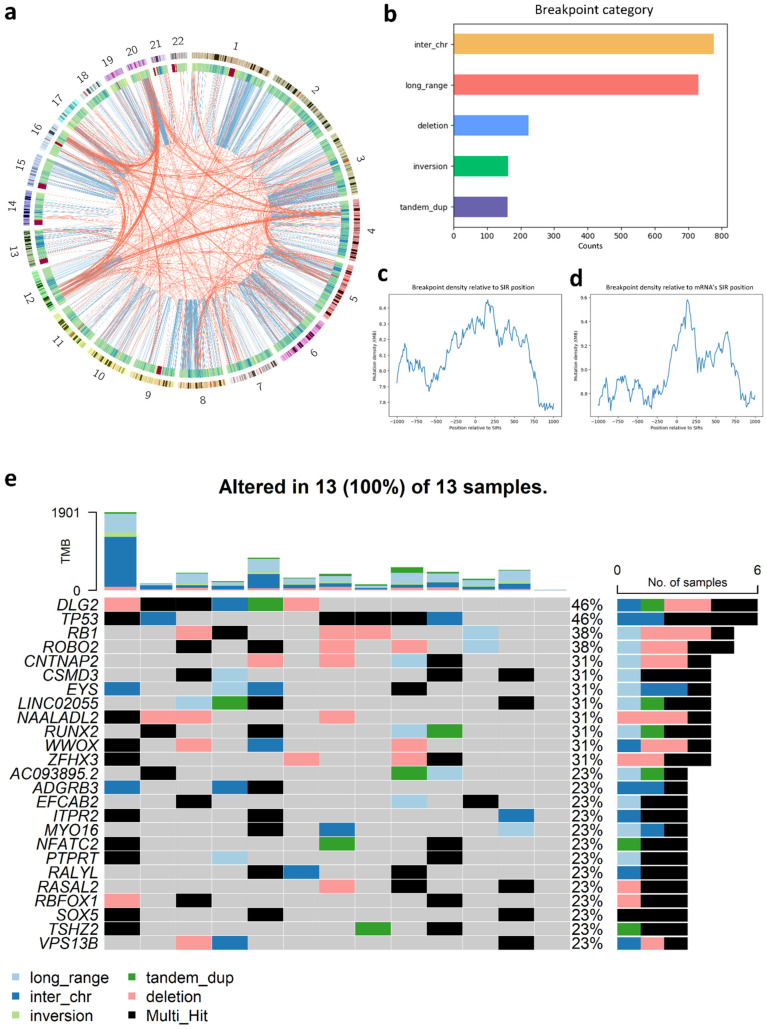
Genome-wide distribution and characterization of SIR-associated breakpoints in osteosarcoma (OS). (**a**) Circos plot showing the chromosomal distribution of SIR-associated intra- and inter-chromosomal breakpoints across the genome; (**b**) Proportion of SIR-associated breakpoints categorized as inter-chromosomal, long-range intra-chromosomal, deletion, inversion, or tandem duplication; (**c**) Breakpoint density profile relative to the center of SIRs and surrounding flanking sequences; (**d**) Breakpoint density profile for SIRs located within protein-coding genes; (**e**) Top 25 genes most frequently affected by SIR-associated breakpoints across 13 OS patients. Bars represent the number of patients with mutations in each gene.

From the 3216 somatic breakpoints detected in 13 OS patients, 2056 (63.9%) overlapped SIR regions. Genome-wide distribution analysis (Figure 10a) revealed distinct chromosomal patterns: chromosomes 1, 3, 6, 8, and 15 exhibited pronounced enrichment of intra-chromosomal breakpoints, whereas chromosomes 4, 5, 12, 16, and 20 showed a higher prevalence of inter-chromosomal breakpoints. Classification of breakpoint types (Figure 10b) indicated that most SIR-associated breakpoints were inter-chromosomal or long-range intra-chromosomal with fewer classified as deletions, inversions, and tandem duplications.

Breakpoint density profiling relative to SIR coordinates exhibited a clear enrichment near, but not exactly at, the SIR center (Figure 10c), with a stronger peak observed in SIRs located within protein-coding genes (Figure 10d). These patterns suggest that SIRs, particularly those embedded in coding regions, may contribute to local genomic instability and structural variation in OS.

Gene-level analysis identified the top 25 genes with the highest frequency of SIR-associated breakpoints (Figure 10e). A substantial fraction were tumor suppressors, including *TP53*, *RB1*, *CNTNAP2*, *ROBO2*, *CSMD3*, *ZFHX3*, and *PTPRT*. Notably, *TP53* and *RB1* were altered in 46% and 38% of patients, respectively, underscoring their pivotal roles in OS pathogenesis. Interestingly, *TP53*, often categorized as either a tumor suppressor gene or oncogene depending on mutation context [72], was identified alongside *NFATC2* as a recurrent oncogene affected by SIR-associated breakpoints. Two lncRNAs, *LINC02055* (31%) and *AC093895.2* (23%), were also among the most frequently disrupted genes, highlighting the potential impact of lncRNA dysregulation on OS development.

**Figure 11:**
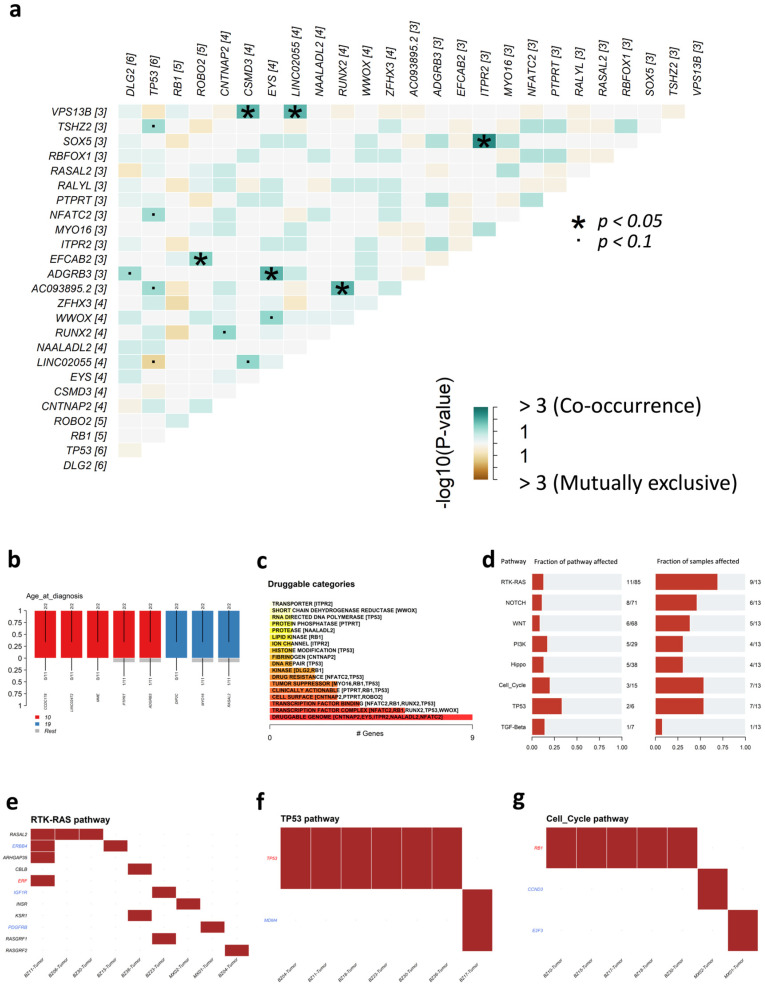
Co-occurrence patterns, clinical enrichment, druggability, and oncogenic pathway alterations of SIR-associated breakpoint genes in osteosarcoma (OS). (**a**) Co-occurrence and mutual exclusivity analysis among the top 25 breakpoint-affected genes (Fisher’s exact test); (**b**) Age-at-diagnosis enrichment analysis for genes harboring SIR-associated breakpoints; (**c**) Drug–gene interaction annotation for the top mutated genes, grouped into druggable categories; (**d**) Oncogenic pathways altered by genes with SIR-associated breakpoints, ranked by frequency; (**e**–**g**) Detailed pathway alteration maps for the RTK–RAS, TP53, and cell cycle pathways, respectively, showing the fraction of patients affected. Gene names are color-coded: blue indicates oncogenes and red indicates tumor suppressor genes.

Co-occurrence analysis (Figure 11a) revealed extensive positive associations among breakpoint-affected genes, such as *TP53* with *NFATC2*, and *LINC02055* with *VPS13B*. Only one mutually exclusive relationship was detected, between *LINC02055* and *TP53*, suggesting potential pathway dependence. Clinical enrichment analysis (Figure 11b) showed age-specific mutation patterns: *CCDC178*, *LINC02472*, *MME*, *PTPRT*, and *ADGRB3* were enriched in patients diagnosed at age 10, while *DIP2C*, *MYO16*, and *RASAL2* were enriched in those diagnosed at age 19. Drug-gene interaction analysis (Figure 11c) indicated that many of the top mutated genes belong to druggable categories such as the druggable genome, cell surface, ion channels, and transcription factor complexes.

Pathway-level analysis (Figure 11d–g) revealed frequent alteration of oncogenic pathways. The RTK-RAS pathway was the most affected, with 11 genes altered in 9 of 13 patients. TP53 and cell cycle pathways were also disrupted in over half of the cohort. Collectively, these findings suggest that SIRs may serve as structural instability hotspots, driving breakpoints that disrupt tumor suppressors, oncogenes, and key oncogenic signaling pathways in OS.

## 4. Discussion

In this study, we systematically characterized SIRs in the human genome and investigated their mutational profiles across multiple cancer types, with a particular focus on osteosarcoma. Our analysis revealed several key findings: first, SIRs are highly abundant and unevenly distributed across the human genome, showing a preference for low GC content regions and lncRNAs; second, across six common cancers, increased INDEL density within SIR spacer regions is a consistent feature, where elevated SNV and breakpoint density is cancer-type specific; third, in OS, both SNV and INDEL densities are significantly enriched in SIR spacer regions; fourth, genomic regions with higher SIR density tend to accumulate more SNV and INDEL mutations; and fifth, SIR-associated mutations frequently occur in functionally important protein-coding genes and lncRNAs, potentially converging on DNA repair and oncogenic signaling pathways.

### 4.1. General Mutational Hotspot Properties of SIRs

Our findings support the notion that SIRs act as mutational hotspots, particularly within their spacer regions. This is consistent with previous reports in breast cancer showing increased mutation density in SIR spacers, likely due to hairpin and cruciform structure formation during DNA transcription or replication that induces DNA double-strand breaks [16,18,19,20]. The enrichment of INDELs across all six cancers examined suggests that spacer instability may be a general feature of SIR mutagenesis, whereas SNV enrichment may depend on additional, cancer-specific factors such as mutagenic environment or DNA repair deficiencies.

### 4.2. Osteosarcoma-Specific Patterns and Potential Driver Roles

In OS, the simultaneous elevation of both SNV and INDEL densities within SIR spacers suggests a heightened vulnerability of these sequences to multiple forms of mutational damage. Moreover, the positive correlation between SIR density and local mutation burden indicates that genomic regions rich in SIRs may serve as central points for broader genome instability. This is particularly relevant in OS, where chromothripsis and complex rearrangements are frequent [42,43]. The presence of SIR-associated mutations in key DNA damage response genes, such as TP53 and RB2, raises the possibility that SIR-induced instability could exacerbate defects in genome maintenance pathways, creating a vicious cycle that accelerates tumor evolution.

Our analysis identified numerous SIR-associated mutations in oncogenes (e.g., *MECOM*, *NFATC2*) and tumor suppressor genes (e.g., *LRP1B*, *PTPRD*), as well as in cancer-associated lncRNAs (e.g., *MALAT1*, *NEAT1*). Disruption of these genes can alter transcriptional regulation [68,73], cell cycle control [74], and chromatin organization [75], all of which are crucial for OS pathogenesis. The pathway enrichment results suggest that SIR-associated mutations converge on a limited set of core signaling and DNA repair pathways, amplifying their oncogenic potential.

The resemblance of SIR-associated mutational patterns to COSMIC signatures indicative of DNA mismatch repair and double-strand break repair suggests that defective DNA repair processes may act synergistically with SIR-induced DNA double-strand breaks to promote mutagenesis. In this context, SIRs could serve as structural “weak spots” that are particularly prone to breakage when repair pathways are compromised, as is often the case in OS.

Our study highlights two complementary aspects of SIR-associated mutagenesis. First, SIRs represent intrinsic hotspots of genome instability, largely due to their ability to form secondary structures such as hairpins and cruciforms. This general property explains why SIR-associated SNVs and INDELs are consistently enriched across multiple cancer types. At the same time, our OS analysis suggests that not all SIR mutations are random passenger events. In OS, SIR-associated mutations are preferentially located in key cancer driver genes, including *TP53*, *RB1*, *MECOM*, and *PTPRD*, and show associations with specific clinical subtypes, mutational signatures, and oncogenic pathways. These patterns indicate that while the mutability of SIRs is a general genomic landscape effect, certain SIR-linked mutations may be selectively retained and act as putative drivers in OS pathogenesis. Nevertheless, we acknowledge that distinguishing between background mutability and true driver events requires functional validation and larger cohorts, which will be an important direction for future research.

### 4.3. Clinical and Therapeutic Relevance

Given the abundance, structural properties, and preferential localization to functionally important genomic regions, SIRs may represent novel biomarkers for genome instability in OS. Furthermore, the identification of recurrent SIR-associated mutations in actionable genes raises the possibility of incorporating SIR mutational profiling into precision oncology strategies.

While our study provides a comprehensive characterization of SIR mutational landscapes in OS, several limitations should be addressed in future research. First, the relatively small OS cohort analyzed here limits the ability to detect low-frequency SIR-associated events. Second, functional validation is needed to directly link SIR structural dynamics to mutagenesis and tumor progression in OS. Finally, it will be important to assess whether SIR mutational patterns can serve as predictive biomarkers for therapeutic response in clinical settings. In addition to SIRs, repetitive elements such as transposable elements and microsatellites can also contribute to genome instability through mechanisms such as non-allelic homologous recombination and replication slippage [76,77,78]. Future studies may compare mutational landscapes across different repetitive elements to further delineate their relative contributions to genome instability and tumorigenesis.

In conclusion, our work identifies SIRs as pervasive mutational hotspots in the OS genome, implicates them in driving local and global genome instability, and highlights their potential as biomarkers and therapeutic targets. These findings expand our understanding of the structural determinants of mutagenesis in cancer and provide a foundation for future studies aimed at exploiting SIR-associated vulnerabilities in OS treatment.

## Figures and Tables

**Table 1:** The top 10 abundant spacers with varying lengths. Spacers are ranked in descending order by their counts.

Spacer (4 nt)	Total Counts	Spacer (5 nt)	Total Counts	Spacer (6 nt)	Total Counts	Spacer (7 nt)	Total Counts	Spacer (8 nt)	Total Counts
AGTG	26,165	TCTTG	17,167	AGCCAC	22,180	TATATCT	9676	AAGGAAAA	3374
CACT	25,931	CAAGA	16,742	GTGGCT	22,152	AGATATA	9599	TTTTCCTT	3302
AAAA	22,020	AACAG	12,221	GTGGCA	8829	TTTGTAG	8677	GCACTATT	2378
TTTT	22,005	TCTCG	11,594	TGCCAC	8816	CTACAAA	8552	AATAGTGC	2330
TATT	12,522	CGAGA	11,582	CACCAC	8218	TTTGCAG	4247	CGGGAATA	1685
AATA	12,469	TTTTT	7657	GTGGTG	8129	CTGCAAA	4201	GTGTGTGT	1454
CCTC	11,573	AAAAA	7341	TTCTTT	4927	CTCAGTA	4180	ACACACAC	1437
GAGG	11,493	TTTGT	7219	AAAGAA	4917	AGAAATA	3742	TGCAAGAG	1400
ATAA	11,450	TGTTC	6891	CGCCAC	3952	AATTAGG	3714	AAAAAAAA	1169
TTAT	11,209	GAACA	6822	GTGGCG	3925	CCTAATT	3693	TTTTTTTT	1138

## Data Availability

The raw whole genome sequencing data and associated clinical data were obtained from dbGaP (accession: phs000699.v1.p1). The original contributions presented in this study are included in the article/Supplementary Material. Further inquiries can be directed to the corresponding author.

## Supplementary Materials

The following supporting information can be downloaded at https://www.mdpi.com/article/10.3390/genes16101202/s1: Table S1: Number of SIRs within protein coding genes and lncRNAs regions; Table S2: Hypermutated SIRs with significantly higher SNV densities within spacers than in flanking control sequences; Table S3: Hypermutated SIRs with significantly higher INDEL densities within spacers than in flanking control sequences.

## Abbreviations

SIR	Short Inverted Repeat
OS	Osteosarcoma
SNV	Single Nucleotide Variant
INDEL	Small Insertion and Deletion
DSB	Double-Strand Break
HR	Homologous Recombination
NHEJ	Non-Homologous End-Joining
WGS	Whole Genome Sequencing
